# Neuroimaging-Guided Transcranial Magnetic Stimulation for Obsessive-Compulsive Disorder: A Systematic Review and Meta-Analysis

**DOI:** 10.7759/cureus.117561

**Published:** 2026-10-06

**Authors:** Maria Alejandra Rodriguez Gaviria, Paula Andrea Cuenca Cuevas, Rodrigo Nava Muñoz, Angie Coraline Moreno Báez, Andrés Felipe Cardenas Gamboa, Natalia Vanegas Arias, Christopher Alejandro Jiménez Beltrán, Leidy Catherine Guerrero Pasijojoa

**Affiliations:** 1 Medicine, Universidad El Bosque, Bogota, COL; 2 Medicine, Fundación Universitaria Navarra - Uninavarra, Neiva, COL; 3 Radiology, Hospital Ángeles Mocel, Ciudad de México, MEX; 4 Medicine, Colsanitas, Bucaramanga, COL; 5 Medicine, Hospital San José del Guaviare, Guaviare, COL; 6 Medicine, Corporación Universitaria Rafael Nuñez, Santiago de Chile, CHL; 7 Public Health, Universidad Autónoma de Guadalajara, Guadalajara, MEX; 8 Medicine, Universidad Cooperativa de Colombia, Pasto, COL

**Keywords:** a systematic review, navigated transcranial magnetic stimulation, obsessive-compulsive disorder, personalized targeting, resting-state functional connectivity

## Abstract

Rates of response to repetitive transcranial magnetic stimulation (rTMS) in obsessive-compulsive disorder (OCD) vary widely. This systematic review and meta-analysis evaluated whether neuroimaging-guided targeting personalization, from fixed-coordinate neuronavigation to individually connectivity-personalized targeting, moderates clinical response, safety, and neural mechanisms in OCD. Following PRISMA (Preferred Reporting Items for Systematic Reviews and Meta-Analyses) 2020 guidelines, PubMed, the Cochrane Library, and ScienceDirect were searched through July 2026 for randomized and non-randomized studies of neuroimaging-guided transcranial magnetic stimulation (TMS) in OCD. Risk of bias (RoB) was assessed using Cochrane RoB2 for RCTs (randomized controlled trials) and ROBINS-I (Risk of Bias in Non-randomized Studies of Interventions) for non-randomized studies. Random-effects inverse-variance and risk-ratio models pooled Yale-Brown Obsessive Compulsive Scale (Y-BOCS) scores and responder rates; non-poolable data were synthesized narratively. Nine study cohorts across eight publications met inclusion criteria (five RCTs and four open-label/single-arm). Pooled analysis of four sham-controlled RCTs showed no significant benefit of neuroimaging-guided TMS on Y-BOCS total score (MD = −0.64, 95% CI = −3.60, 2.32) or responder rate (RR = 1.46, 95% CI = 0.68, 3.15), with moderate heterogeneity. However, trials using individually connectivity-personalized pre-supplementary motor area (pre-SMA) targeting consistently trended toward benefit, while fixed-coordinate neuronavigated trials trended toward null or sham-favoring results. A narrative synthesis of non-poolable and non-randomized studies showed larger, mostly positive within-group reductions in Y-BOCS scores (25%-48%), particularly among patients with moderate disease severity, alongside mechanistic evidence linking individualized connectivity-based targets to symptom improvement. TMS was well tolerated overall, with mild, transient headache being the most common adverse event and no serious safety concerns identified. Overall, neuroimaging-guided TMS showed no pooled benefit in OCD; however, individually connectivity-personalized targeting may be more beneficial than fixed-coordinate approaches, warranting adequately powered trials that directly compare targeting-personalization strategies.

## Introduction and background

The chronic and debilitating mental illness known as obsessive-compulsive disorder (OCD) is typified by intrusive thoughts (called obsessions) and repetitive behaviors (called compulsions) that sufferers feel driven to carry out [1]. OCD frequently results in severe distress and functional impairment [1]. According to epidemiological statistics, OCD affects 1%-3% of people worldwide, has a lifetime incidence of about 2.3% among adults in US populations, and usually first appears in late adolescence or early adulthood [2-4]. OCD patients typically maintain awareness of the irrationality of their symptoms but are unable to control them; this difference sets OCD apart from many other anxiety-spectrum disorders and presents a significant therapeutic barrier [5]. Although the pathophysiology of OCD is still unclear, neuroimaging and neuropsychological research have repeatedly linked dysfunction in cortico-striato-thalamo-cortical (CSTC) circuits, specifically in the orbitofrontal cortex (OFC), anterior cingulate cortex (ACC), supplementary motor area, and basal ganglia [6-8]. Crucially, the exact location and connection of these defective nodes differ significantly between people; neuroimaging has been crucial in identifying this variability, which fixed anatomical or scalp-landmark targeting cannot, by definition, capture [6].

Pharmacotherapy, primarily selective serotonin reuptake inhibitors (SSRIs), and psychotherapy, specifically cognitive-behavioral therapy (CBT) with exposure and response prevention (ERP), are standard treatment approaches for OCD [9-11]. About 30%-40% of patients do not have sufficient symptom alleviation despite these tried-and-true approaches, highlighting the need for additional or alternative therapy [12,13]. Repetitive transcranial magnetic stimulation (rTMS) holds a unique place among neuromodulation techniques investigated for treatment-resistant OCD, including deep brain stimulation, vagus nerve stimulation, transcranial direct current stimulation, and electroconvulsive therapy, because of its non-invasive profile [14].

Transcranial magnetic stimulation (TMS) broadly refers to the non-invasive application of pulsed magnetic fields to induce focal electrical currents in the underlying cortex [15]. Conventional rTMS uses a figure-eight coil delivering pulses at a fixed frequency over a selected cortical target, whereas deep TMS (dTMS) uses H-coils to stimulate broader and deeper cortical and subcortical volumes [16]. Thus, dTMS is primarily a hardware variant of rTMS, sharing frequency-dependent effects, with high-frequency stimulation generally facilitatory and low-frequency stimulation (typically ≤1 Hz) generally inhibitory [17]. Theta-burst stimulation (TBS) delivers 50-Hz triplet bursts repeated at 5 Hz, allowing shorter sessions; intermittent TBS (iTBS) is generally facilitatory, whereas continuous TBS (cTBS) is generally inhibitory [18]. The H7 coil has FDA clearance as an adjunctive treatment for adults with treatment-resistant OCD after at least one adequate medication trial [19]. TMS in OCD has predominantly included adults aged approximately 18-70 years [20]. Common contraindications include non-removable ferromagnetic material near the head, cochlear implants, and incompatible implanted electronic devices [21]. Seizure history is generally considered a precaution requiring risk-benefit assessment. Adverse effects are usually mild and transient, particularly headache and scalp discomfort; seizures are rare [22]. Neuroimaging-guided targeting ranges from verifying group-derived coordinates using individual anatomy to connectivity-personalized targeting based on each patient's functional-connectivity profile [23].

Mechanistically, rTMS modulates cortical excitability in a frequency-dependent manner by delivering focal electrical currents to targeted cortical regions via rapidly alternating magnetic pulses [15]. Higher frequencies produce facilitatory effects, while low-frequency stimulation (usually 1 Hz) is generally inhibitory [24,25]. Based mostly on the pivotal study by Carmi et al., the field achieved a significant milestone in 2018 when the FDA approved dTMS utilizing the H7 coil, which targets the ACC and medial prefrontal cortex (mPFC) as an adjunct treatment for adults with treatment-resistant OCD [26].

Clinical research examining rTMS effectiveness in OCD has shown noticeably inconsistent results despite this regulatory milestone [27,28]. How the stimulation location is chosen is a source of this heterogeneity that has received very little systematic research; this subject spans a spectrum of growing image-based individualization. On the one hand, coil placement may be based on set group-level coordinates or scalp-based anatomical landmarks, which can only be confirmed by neuronavigation software that tracks a standard target rather than a patient-specific one. MRI-guided structural neuronavigation using each patient's unique T1-weighted anatomy, functional MRI (fMRI)-guided task-based functional localization of the target region, and, at the most individualized end, resting-state connectivity-guided targeting, in which the stimulation site is directly derived from an individual's functional-connectivity map to a hypothesized network hub, are examples of increasingly more customized methods [29]. Dehghani-Arani et al. (2024) discovered that the magnitude of rTMS-induced electrical fields within targeted circuits, rather than the nominal stimulation site alone, predicted clinical response across 27 randomized controlled trials (RCTs) [30]. This finding suggests that targeting precision, rather than just anatomical region or stimulation frequency, may be a significant driver of outcome. Whether this personalization gradient translates into measurable clinical benefit in OCD specifically, however, has not been formally tested in the existing meta-analytic literature.

Three recent reviews illustrate how existing syntheses of rTMS for OCD have organized evidence by stimulation frequency and anatomical target, while leaving targeting methodology unexamined. Fitzsimmons et al. conducted a pairwise and network meta-analysis of 21 RCTs clustered by frequency-location combinations, identifying efficacious protocols without regard to whether the coil was neuronavigated, connectivity-personalized, or freehand-positioned [31]. Liu et al. narrowed the lens to non-neuronavigated, low-frequency surface-coil protocols specifically, finding no significant benefit over sham (MD = −0.69, 95% CI = −1.75, 0.38) and explicitly noting that "whether neuronavigation and cortical field verification can bridge this performance gap requires adequately powered trials designed for direct protocol comparison," a question their study excluded by definition [32]. Khosravi et al., the broadest of the three at 47 RCTs, gesture toward neuroimaging-based personalization as a promising future direction but treat it narratively rather than as a formally tested moderator [14]. To our knowledge, prior reviews formally distinguishing individually connectivity-personalized targeting from standard-coordinate targeting merely verified by neuronavigation are rare, a distinction that tracks a consistent split in treatment response across trials. This leaves clinicians without evidence on whether the degree of imaging-based individualization, rather than target region or frequency alone, drives clinical benefit in treatment-resistant OCD. Therefore, this review aimed to systematically evaluate the efficacy, safety, and underlying neural mechanisms of neuroimaging-guided TMS for OCD and specifically to determine whether the degree of targeting personalization, ranging from fixed-coordinate neuronavigation to individually connectivity-personalized targeting, moderates clinical response, as measured by Y-BOCS total score, responder/remission rate, and secondary clinical and neurocognitive outcomes.

## Review

Methods

Methodology

This systematic review was not registered with PROSPERO (International Prospective Register of Systematic Reviews), which is acknowledged as a limitation. It was, however, conducted in accordance with the Preferred Reporting Items for Systematic Reviews and Meta-Analyses (PRISMA) 2020 guidelines [33].

Eligibility Criteria

Eligibility was defined using a PICOS (Population, Intervention, Comparator, Outcome, Study design) framework. Eligible populations comprised adults (≥18 years) with a primary DSM (Diagnostic and Statistical Manual of Mental Disorders)- or ICD (International Classification of Diseases)-diagnosed OCD. Eligible interventions included any TMS modality, conventional rTMS, dTMS (H-coil), iTBS, or cTBS, in which the coil target was determined using neuroimaging. Four targeting approaches were considered eligible: (a) MRI-guided structural neuronavigation using the patient's own T1-weighted scan; (b) fMRI-guided task-based functional localization; (c) resting-state connectivity-guided targeting, in which the stimulation site was individually derived from the patient's own functional-connectivity map; and (d) neuronavigation-guided targeting more broadly, in which an image-based coil-tracking system was used to set or verify coordinates, even where the coordinate itself represented a standard group-level target applied via imaging rather than scalp-landmark or "5-cm rule" placement. Studies in which neuroimaging was used only to characterize, predict, or explain outcomes after stimulation at a scalp-landmark or standard anatomical coordinate (i.e., imaging-as-predictor rather than imaging-as-targeting) were excluded from the primary pool. Beyond the imaging-as-predictor exclusion above, records were also excluded if they enrolled patients whose primary diagnosis was not OCD (e.g., OCD as a secondary or comorbid diagnosis only), used a TMS target selected exclusively via scalp-landmark or fixed anatomical ("5-cm rule") coordinates with no imaging input at any stage, were single-patient case reports, were conference abstracts without a linked full-text article, or were animal/preclinical studies.

Eligible comparators included sham/placebo TMS, waitlist, or active comparator targets. The primary outcome was the Yale-Brown Obsessive Compulsive Scale (Y-BOCS) total score [34]; secondary outcomes included responder/remission rate, Clinical Global Impression (CGI) scores [35], and secondary mood/anxiety measures. RCTs were eligible for inclusion in the quantitative meta-analytic pool; open-label studies, case series, and retrospective cohorts were eligible for inclusion in a separate narrative/sensitivity synthesis but were not pooled quantitatively with RCT data. Only studies published in English were eligible for inclusion; non-English-language publications were excluded regardless of other eligibility criteria.

Information Sources and Search Strategy

A systematic search was conducted across PubMed, the Cochrane Library, and ScienceDirect from database inception through July 14, 2026, using database-specific syntax adapted to each platform's controlled vocabulary and field-search capabilities. The search strategy is given in the Appendix table.

In addition to the database searches, the reference lists of all included studies and relevant prior systematic reviews and meta-analyses were manually screened to identify any additional eligible studies not captured by the electronic search.

Study Selection and Data Extraction

Records identified through the database searches and reference screening were exported into EndNote (Clarivate, Philadelphia, PA), where duplicates were removed. Titles and abstracts, followed by full texts, were then screened manually by two independent reviewers against the eligibility criteria, with disagreements resolved by discussion or a third reviewer. Data were extracted independently and in duplicate by two reviewers using a standardized extraction form capturing study design, population characteristics, sample size, mean age, targeting methodology, stimulation parameters (frequency, intensity, and pulses per session), control condition, treatment schedule, and outcome data (means, standard deviations, and dichotomous responder counts where available).No automated data-extraction, text-mining, or large-language-model tool was used at any stage of screening or extraction; all steps were performed manually by the reviewers described above.

Risk of Bias Assessment

Risk of bias in the five RCTs was independently assessed by two reviewers using the Cochrane Risk of Bias 2 (RoB2) tool across five domains: bias arising from the randomization process, bias due to deviations from intended interventions, bias due to missing outcome data, bias in measurement of the outcome, and bias in selection of the reported result [36]. Risk of bias in the four non-randomized studies was assessed using the Risk of Bias in Non-randomized Studies of Interventions (ROBINS-I) tool across seven domains: bias due to confounding, bias due to selection of participants, bias in classification of interventions, bias due to deviations from intended interventions, bias due to missing data, bias in measurement of outcomes, and bias in selection of the reported result [37]. Disagreements between reviewers were resolved by consensus.

Data Synthesis and Statistical Analysis

Where at least three RCTs reported extractable means and standard deviations (or dichotomous event counts) for a given outcome, data were pooled using a random-effects, inverse-variance model for continuous outcomes (Y-BOCS total score) and a random-effects risk ratio (RR) model for dichotomous outcomes (responder rate), given anticipated clinical heterogeneity across targeting methods using Review Manager (RevMan) version 5.4 (The Cochrane Collaboration, London, UK). Between-study heterogeneity was quantified using Tau², the χ² test, and the I² statistic. Findings from studies that could not be statistically pooled, due to reporting only percentage change, figure-derived values, or omnibus test statistics rather than extractable group means, and findings from all non-randomized studies were synthesized narratively, organized by outcome domain (primary Y-BOCS outcome, responder/remission rate, secondary clinical outcomes, neurocognitive outcomes, neuroimaging/connectivity mechanistic findings, and adverse events/tolerability). Publication bias was not formally assessed via funnel plot or Egger's/Begg's test because each pooled outcome (Y-BOCS total score and responder rate) included only four studies, well below the approximately 10 studies generally considered the minimum for such tests to be interpretable.

Results

A total of 390 records were identified through database searching, including 185 from PubMed, 126 from the Cochrane Library, and 79 from ScienceDirect. After removing 52 duplicate records, 338 unique records remained for title and abstract screening. Of these, 293 records were excluded based on the predefined eligibility criteria. The full texts of 45 articles were assessed for eligibility, of which 37 studies were excluded due to incompatible study design (n = 10), incompatible outcomes (n = 9), incompatible intervention (n = 9), unclear methodologies (n = 8), or unavailable full text (n = 1). Ultimately, eight studies met the inclusion criteria and were included in the systematic review (Figure 1).

**Figure 1:**
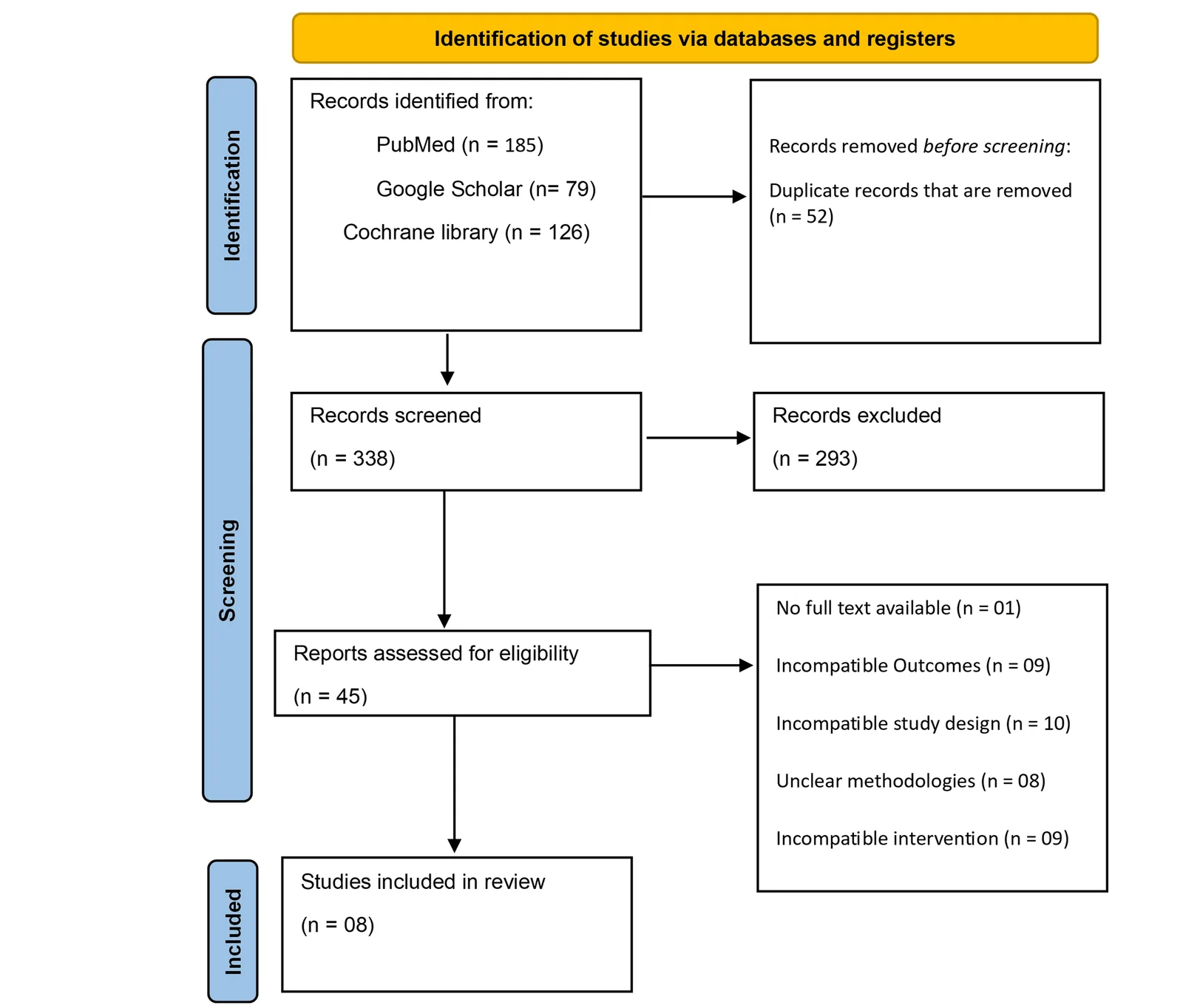
PRISMA flow diagram PRISMA: Preferred Reporting Items for Systematic Reviews and Meta-Analyses.

Study Selection and Characteristics

Nine study cohorts across eight publications met the inclusion criteria for this review, as Ni et al. reported two independent cohorts, a preliminary inpatient randomized trial and a separate outpatient extension study, within a single article [38]. Five sham-controlled randomized trials were identified [38-42], along with four open-label or single-arm studies [38,43-45]. Targeting methodology varied along a personalization gradient: two trials used individually connectivity-personalized pre-supplementary motor area (pre-SMA) targets derived from resting-state functional connectivity to the subthalamic nucleus [38,40]; two used standard-coordinate or structurally neuronavigated pre-SMA targets [39,42]; one used individually functionally localized bilateral SMA [43]; two used deep double-cone coil stimulation of the dorsal ACC/medial prefrontal cortex [41,44]; and one used sequential neuronavigated stimulation of the dorsolateral prefrontal cortex (DLPFC) and OFC (Tables 1, 2) [45].

**Table 1:** Characteristics of the studies included RCT: Randomized controlled trial; OCD: Obsessive-compulsive disorder; rTMS: Repetitive transcranial magnetic stimulation; cTBS: Continuous theta-burst stimulation; iTBS: Intermittent theta-burst stimulation; TBS: Theta burst stimulation; SD: Standard deviation; SEM: Standard error of the mean; F: Female; M: Male; pre-SMA: Pre-supplementary motor area; SMA: Supplementary motor area; DLPFC: Dorsolateral prefrontal cortex; R: Right; L: Left; OFC: Orbitofrontal cortex; mPFC: Medial prefrontal cortex; dACC: Dorsal anterior cingulate cortex; BA: Brodmann area; STN: Subthalamic nucleus; rsFC: Resting-state functional connectivity; FC: Functional connectivity; BOLD: Blood-oxygen-level-dependent; MRI: Magnetic resonance imaging; MNI: Montreal Neurological Institute; EMG: Electromyography; RMT: Resting motor threshold; Hz: Hertz; DSM-5: Diagnostic and Statistical Manual of Mental Disorders, 5th Edition.

Author, Year	Study Design	Population (N)	Mean Age (SD)	Sex	Target Region	Targeting Method	Intervention	Control	Treatment Schedule
Pelissolo et al., 2016 [42]	RCT, double-blind, sham-controlled, parallel-group (three centers, France)	Adults, treatment-resistant OCD; 40 randomized (rTMS n = 20/Sham n = 20); 36 analyzed (n = 20/16); 33 completers	rTMS 39.1 (10.4); Sham 42.3 (10.6)	23F/17M overall	Bilateral pre-SMA	MRI-guided structural neuronavigation to fixed anatomical landmark (anterior commissure plane)	1 Hz rTMS, 100% RMT, 1500 pulses/session	Sham coil (mu-metal shield)	20 sessions, 5 days/week × 4 weeks; follow-up at week 12
Ni et al., 2024 (Preliminary) [38]	RCT, sham-controlled, single-center (inpatients)	Adult OCD inpatients; n = 50 randomized (cTBS n = 23/Sham n = 22)	cTBS 26.61 (6.99); Sham 28.55 (8.97)	cTBS 16M/7F; Sham 15M/7F	Right pre-SMA (personalized)	Individualized rsFC-guided targeting (pre-SMA voxel with highest FC to STN)	cTBS, 80% RMT, 1800 pulses/session	Sham coil (matched sensation, reduced cortical activity)	10 sessions/day × 5 consecutive days
Ni et al., 2024 (Extension) [38]	Open-label, single-arm, external validation (outpatients)	Adult OCD outpatients, lower baseline severity; n = 32	27.25 (8.00)	16M/16F	Right pre-SMA (personalized)	Same rsFC-guided method as preliminary experiment	cTBS, 80% RMT, 1800 pulses/session	None (single-arm)	1 session/day × 15 consecutive days
Stubbeman et al., 2024 [45]	Retrospective, open-label chart review, single clinic	Adults, moderate-severe OCD, completed ≥20 treatments; n = 14	26.4 (8.14)	8M/6F	Sequential: R-DLPFC → L-DLPFC → R-OFC	Neuronavigated to fixed Talairach coordinates (3T MRI and ANT-Neuro Visor)	TBS-20Hz (cTBS/iTBS/cTBS sequence)	None (retrospective, no comparator)	5 days/week until remission or discontinuation
Modirrousta et al., 2015 [44]	Open-label, single-arm, single-center (Winnipeg, Canada)	Adults with OCD, no comorbid axis I disorder; n = 10	28.7 (12.1)	Not broken down by count	Bilateral mPFC/dACC (BA24/32)	Individual neuronavigation (BrainSight 2, MNI-normalized, and EMG-anchored)	1 Hz deep rTMS (double-cone coil), 110% RMT, 1200 pulses/session	None (open-label)	10 daily sessions; follow-up at 1 month
Meek et al., 2021 [41]	RCT, double-blind, sham-controlled, single-center (extension of Modirrousta 2015 pilot)	Adults with DSM-5 OCD; 23 enrolled; 20 completers (Active n = 10/Sham n = 10)	Active 45.0 (16.7); Sham 38.3 (11.5)	Active 6F/4M; Sham 4F/6M	dACC/BA32	Individual neuronavigation (BrainSight, BA-mapped, and toe-motor-anchored)	1 Hz rTMS (double-cone coil), 120% RMT, 1200 pulses/session	Sham double-cone coil	20 sessions, 2/day × 2 weeks; follow-up to 3 months
Harika-Germaneau et al., 2019 [39]	RCT, double-blind, sham-controlled, single-center (France)	Treatment-resistant OCD outpatients; n = 30 randomized; 28 analyzed (Active n = 14/Sham n = 14)	Active 46.3 (10.1); Sham 48.2 (12.9)	Not specified by count	Pre-SMA	Individually localized via MRI + neuronavigation (fixed target, not connectivity-personalized)	Active cTBS, 70% RMT, 600 pulses/session	Sham cTBS (flipped coil)	30 sessions, 5/week × 6 weeks; follow-up at week 12
Ji et al., 2021 [40]	RCT, double-blind, sham-controlled (China)	DSM-5 OCD, medication-resistant/naïve; 44 treated (23 real/21 sham); 37 completers (20/17)	Real 27.75 (SEM 1.58); Sham 27.65 (SEM 1.73)	Not specified by count	Right pre-SMA (personalized)	Individualized rsFC-guided (pre-SMA–STN connectivity)	1 Hz rTMS, 110% RMT, 1800 pulses/session	Sham coil	14 consecutive daily sessions
Mantovani et al., 2021 (pilot) [43]	Open-label, uncontrolled pilot (Italy)	DSM-5 OCD; n = 9 enrolled, 8 completers	Enrolled 44.43 (11.10); completers 40.75 (13.24)	Enrolled 5F; completers 4F	Bilateral SMA	Individualized functional localization (rs-BOLD activation, not FC-seed personalized)	1 Hz rTMS, 110% RMT, 3600 pulses/session, 2×/day	None (open-label)	10 sessions, 2/day × 5 days; follow-up at 3 months

**Table 2:** Summary of primary and secondary outcomes by study RCT: Randomized controlled trial; Y-BOCS: Yale-Brown Obsessive Compulsive Scale; MD: Mean difference; CI: Confidence interval; RR: Risk ratio; pre-SMA: Pre-supplementary motor area; SMA: Supplementary motor area; cTBS: Continuous theta burst stimulation; dACC: Dorsal anterior cingulate cortex; BA: Brodmann area; DLPFC: Dorsolateral prefrontal cortex; R: Right; L: Left; OFC: Orbitofrontal cortex; mPFC: Medial prefrontal cortex; d: Cohen's d; t: t-statistic; F: F-statistic; Z: Z-statistic; p: p-value; MADRS: Montgomery-Åsberg Depression Rating Scale; BAI: Beck Anxiety Inventory; BDI/BDI-II: Beck Depression Inventory (2nd edition); HADS: Hospital Anxiety and Depression Scale; CGI-S: Clinical Global Impression – Severity; CGI-I: Clinical Global Impression – Improvement; MOCI: Maudsley Obsessive Compulsive Inventory; OTL: Obsessive Thoughts List; GAF: Global Assessment of Functioning; Y-BOCS-SR: Y-BOCS Self-Report.

Study	Design/Target	Primary Y-BOCS Finding	Responder/Remission Rate	Key Secondary Findings
Harika-Germaneau et al., 2019 [39]	RCT, sham-controlled; standard-coordinate/neuronavigated pre-SMA	MD = 2.14 (95% CI −2.37, 6.65), numerically favoring sham; pooled	RR = 0.80 (95% CI 0.27, 2.37), favoring sham	No significant group differences on MADRS, Brown Assessment of Beliefs Scale, BAI, CGI-severity, and HADS (all p ≥ .264)
Ji et al., 2021 [40]	RCT, sham-controlled; connectivity-personalized pre-SMA	MD = −2.78 (95% CI −6.38, 0.82), favoring active; pooled	RR = 3.83 (95% CI 0.95, 15.34) — strongest effect of the four pooled trials	Connectivity-to-"ideal-target" similarity predicted improvement (r = .60, p = .009) in active, not sham (r = −.004, p = .99); cross-validated in three external cohorts
Ni et al., 2024 (preliminary, inpatient) [38]	RCT, sham-controlled; connectivity-personalized right pre-SMA (cTBS)	MD = −3.36 (95% CI −7.38, 0.66), favoring active; pooled	RR = 2.07 (95% CI 0.96, 4.48)	Obsession subscale Time × Group interaction significant (F = 4.43, p = .04) despite non-significant total score (F = 3.25, p = .08); benefit confined to moderate-severity patients (F = 10.16, p = .004)
Pelissolo et al., 2016 [33]	RCT, sham-controlled; standard-coordinate/neuronavigated bilateral pre-SMA	MD = 2.30 (95% CI −2.23, 6.83), numerically favoring sham; pooled	RR = 0.53 (95% CI 0.10, 2.76), favoring sham	No significant differences on CGI-S (p=.72), CGI-I (p=.54), MOCI, OTL, GAF, MADRS, and BAI (all p = .14–.84)
Meek et al., 2021 [32]	RCT, sham-controlled; deep double-cone dACC/BA32 (not pooled – % change/figure data only)	Significant within-group change, active (F = 5.891, p < .001), not sham (F = 1.529, p = .200); 28.0% (active) vs 11.7% (sham) reduction at 1 month	Not reported as a dichotomous responder rate	Flanker-task reaction time improved in active group only (congruent/incongruent/post-correct trials, all p ≤ .001); obsessive (F=3.702, p=.007) and compulsive (F=4.200, p=.003) subscales improved within active arm only
Ni et al., 2024 (extension, outpatient) [38]	Open-label, single-arm; connectivity-personalized pre-SMA	t = 5.56, p < .001, d = 0.98; concentrated in moderate-severity subgroup (d = 1.08)	50% (16/32)	No significant benefit in mild or severe subgroups after correction for multiple comparisons
Modirrousta et al., 2015 [44]	Open-label, single-arm; deep bilateral mPFC/dACC	F = 63.12, p < .001; mean reduction 39.4% (SD 15.4), sustained at 1-month follow-up	100% (10/10) response among completers (range 23%–76.5% reduction)	Open-label pilot that directly preceded the Meek et al. 2021 sham-controlled replication
Stubbeman et al., 2024 [45]	Retrospective, open-label; sequential neuronavigated R-DLPFC→L-DLPFC→R-OFC theta-burst	48% mean Y-BOCS reduction (Z = 3.08, p = .002, d = −1.8)	Remission (Y-BOCS-SR ≤ 14): 64% (9/14) at mean 6.1 weeks; rising to 65% reduction among remitters	Comorbid depression remission 58% (7/12); anxiety remission 62% (8/13); large within-group BDI-II (d=−1.7), and BAI (d=−1.9) reductions
Mantovani et al., 2021 (pilot) [43]	Open-label, uncontrolled pilot; individually functionally localized bilateral SMA	~25.4% reduction (F [1,7] = 16.162, p = .005)	50% (4/8) at immediate post-treatment; two initial non-responders improved by three-month follow-up	Decreased SMA–basal ganglia/thalamus/cerebellum connectivity post-stimulation; higher baseline severity predicted greater residual connectivity and non-response

Quality Assessment

Risk of bias for the five RCTs was assessed using the Cochrane RoB2 tool (Figure 2). Two studies, Harika-Germaneau et al. and Meek et al., were judged to be at overall low risk of bias across all five domains [39,41]. Ji et al. and Pelissolo et al. were rated as having some concerns overall, driven respectively by moderate risk in the deviations-from-intended-intervention domain (D2) and the missing-outcome-data domain (D3) [40,42]. The preliminary experiment reported by Ni et al. was also judged to have some concerns overall, with moderate risk identified in the randomization process (D1), deviations from intended intervention (D4), and selection of the reported result (D5). No RCT was judged to be at overall high/serious risk of bias [38] (Figure 2).

**Figure 2:**
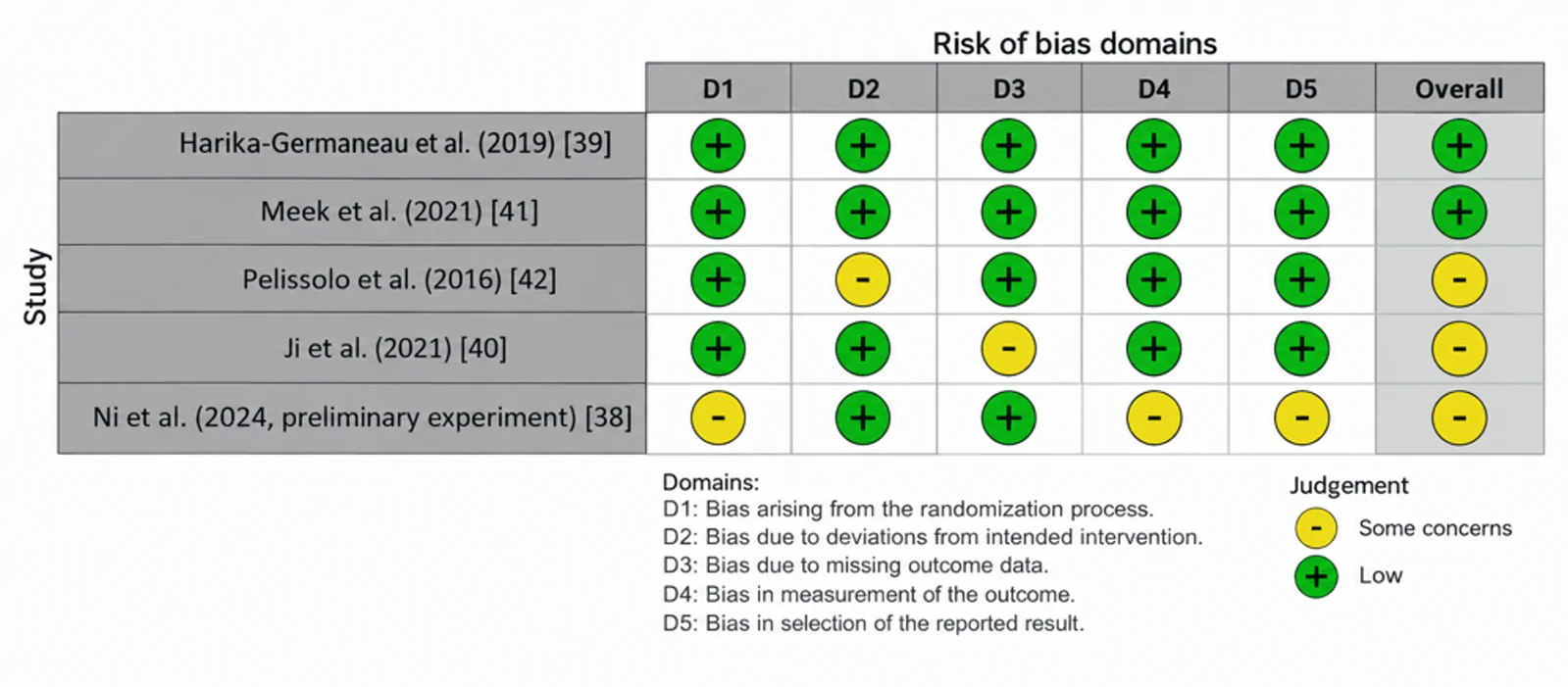
Quality assessment of RCTs by RoB 2.0 RCT: Randomized controlled trials; RoB: Risk of bias.

The four non-randomized studies were assessed using the ROBINS-I tool (Figure 3). All four were judged to be at overall serious risk of bias, primarily driven by uncontrolled confounding (D1), which was rated serious in every study given the absence of a comparator arm or randomization. Stubbeman et al. showed the greatest vulnerability, with additional serious-risk ratings in participant selection (D2) and missing data (D5), alongside serious risk in outcome measurement (D6) [45]. The Ni et al. extension experiment and Mantovani et al. pilot showed comparatively stronger performance on classification of interventions (D3) and deviations from intended interventions (D4), both rated low risk, but were still limited by confounding-related bias inherent to their open-label, single-arm designs [38,43]. Given these serious risk-of-bias ratings, findings from the non-randomized studies were prioritized for narrative synthesis rather than pooled quantitative analysis (Figure 3).

**Figure 3:**
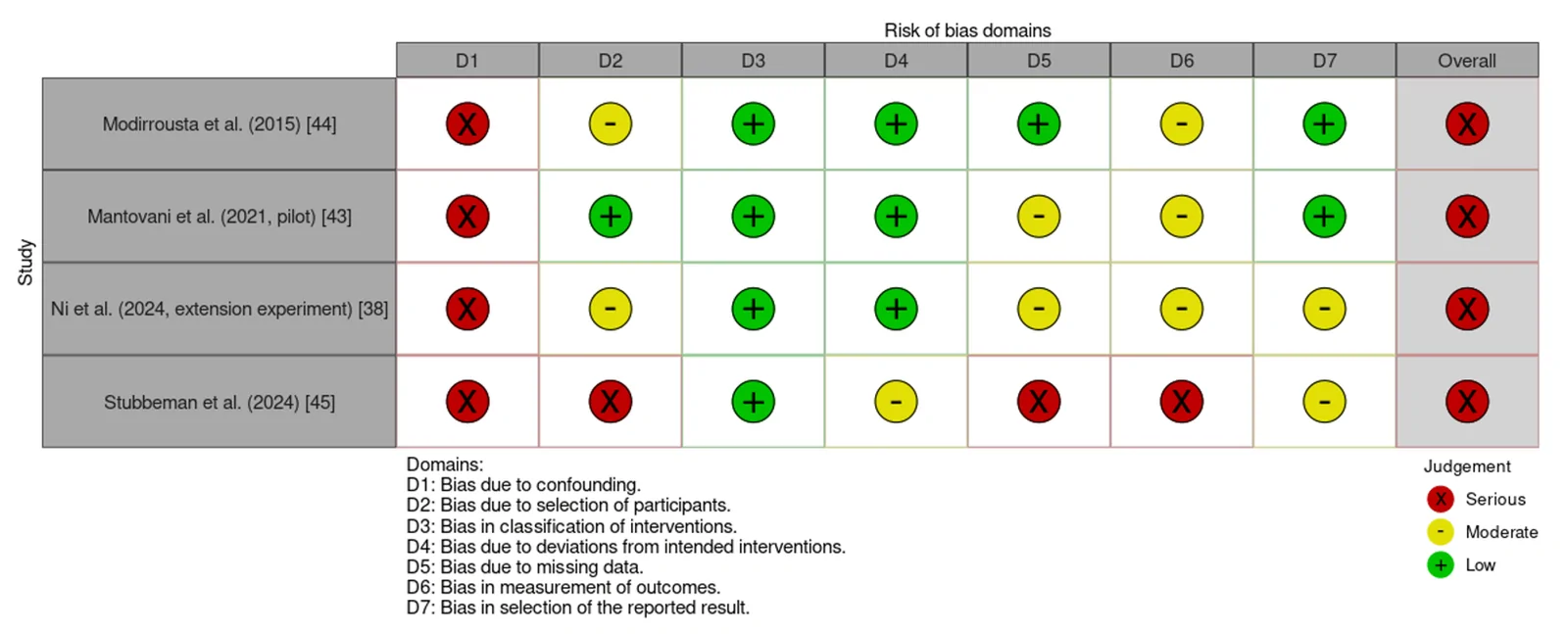
Risk of bias assessment for non-randomized studies by ROBINS-I ROBINS-I: Risk of Bias in Non-randomized Studies of Interventions.

Y-BOCS Total Score

Meta-analysis of RCTs: Four sham-controlled trials contributed endpoint Y-BOCS means and standard deviations suitable for pooling: Harika-Germaneau et al., Ji et al., Ni et al. (preliminary experiment), and Pelissolo et al., totaling 77 participants in the experimental arms and 69 in the control arms [29-31,33]. A random-effects, inverse-variance model produced a pooled mean difference (MD) of −0.64 (95% CI = −3.60, 2.32), indicating no statistically significant overall benefit of neuroimaging-guided TMS over sham (Z = 0.42, p = .67). Individual estimates were directionally inconsistent: Ji et al. [40] and Ni et al. [38] both favored active stimulation (MD = −2.78, 95% CI = −6.38, 0.82; MD = −3.36, 95% CI = −7.38, 0.66), while Harika-Germaneau et al. [39] and Pelissolo et al. [42] numerically favored sham (MD = 2.14, 95% CI = −2.37, 6.65; MD = 2.30, 95% CI = −2.23, 6.83). No individual trial's confidence interval excluded the null, and heterogeneity was moderate (Tau² = 4.66, χ² = 6.14, df = 3, p = .10, I² = 51%), consistent with the divergent targeting strategies underlying these four trials (Figure 4).

**Figure 4:**
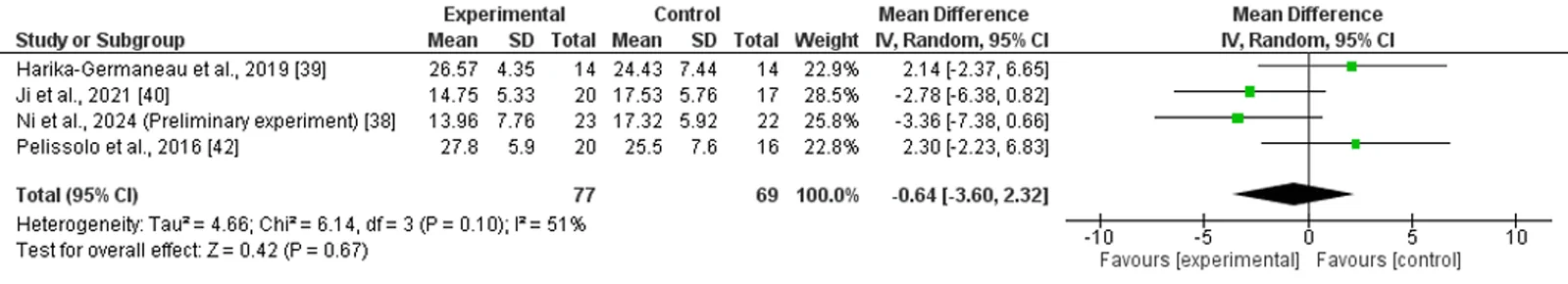
Forest plot of Yale-Brown Obsessive-Compulsive Scale (Y-BOCS)

Narrative synthesis of non-poolable findings: A fifth sham-controlled trial, Meek et al., targeted the dorsal ACC (Brodmann area 32) via double-cone coil but reported only percentage change and figure-based post-treatment values rather than numeric means and standard deviations, precluding pooling [41]. Per-protocol analysis showed a significant within-group Y-BOCS change over time in the active arm (F = 5.891, p < .001) but not sham (F = 1.529, p = .200), with peak improvement at one-month follow-up (28.0% active vs. 11.7% sham). This trial directly extended an earlier open-label pilot by the same group: Modirrousta et al. treated 10 patients with deep rTMS to the bilateral medial prefrontal cortex/dACC and found a significant Y-BOCS reduction across treatment (63.12, p < .001), averaging 39.4% (SD = 15.4) and sustained at one-month follow-up, a substantially larger uncontrolled effect than the sham-controlled replication, likely reflecting the absence of blinding and placebo control in the pilot design [44].

Three further single-arm studies reported positive Y-BOCS reductions without a comparator. The outpatient extension experiment of Ni et al. applied the same connectivity-personalized pre-SMA protocol used in their randomized trial to an independent, lower-severity sample (n = 32), finding a significant pre-post reduction (t = 5.56, p < .001, d = 0.98) concentrated among moderate-severity patients (d = 1.08), with no significant benefit in mild or severe subgroups after correction for multiple comparisons [38]. Stubbeman et al. reported a 48% mean Y-BOCS reduction (Z = 3.08, p = .002, d = −1.8) across 14 patients receiving sequential TBS to prefrontal and orbitofrontal targets, rising to a 65% reduction among the 64% who achieved remission [45]. Mantovani et al. reported a significant reduction in a small pilot (n = 8; F (1,7) = 16.162, p = .005, ~25.4% reduction), notably with the two non-responders showing the highest baseline severity in the sample, an early signal of the severity-dependent response pattern later formalized by Ni et al. [38,43].

Responder Rate

Meta-analysis of RCTs: The same four randomized trials reported dichotomous responder outcomes (variably defined as ≥25%-40% Y-BOCS reduction) and were pooled using a random-effects risk RR model, comprising 76 experimental and 68 control participants with 28 and 16 responders, respectively (Figure 5). The pooled RR was 1.46 (95% CI = 0.68, 3.15), favoring active stimulation but not reaching statistical significance (Z = 0.97, p = .33). Individual estimates again diverged by targeting method: Ji et al. [40] showed the strongest effect (RR = 3.83, 95% CI = 0.95, 15.34), followed by Ni et al. (RR = 2.07, 95% CI = 0.96, 4.48), both trials using connectivity-personalized pre-SMA targets [38]. In contrast, the two standard-coordinate trials showed responder rates numerically favoring sham: Harika-Germaneau et al. (RR = 0.80, 95% CI = 0.27, 2.37) and Pelissolo et al. (RR = 0.53, 95% CI = 0.10, 2.76) [39,42]. Heterogeneity was moderate (Tau² = 0.26, χ² = 5.24, df = 3, p = .15, I² = 43%). This consistent split, with connectivity-personalized trials trending toward benefit and fixed-coordinate trials trending toward null or sham-favoring results, mirrors the pattern observed for Y-BOCS total score and suggests targeting personalization as a plausible, though not yet formally tested, effect moderator; the current pooled sample is underpowered for subgroup meta-regression (Figure 5).

**Figure 5:**
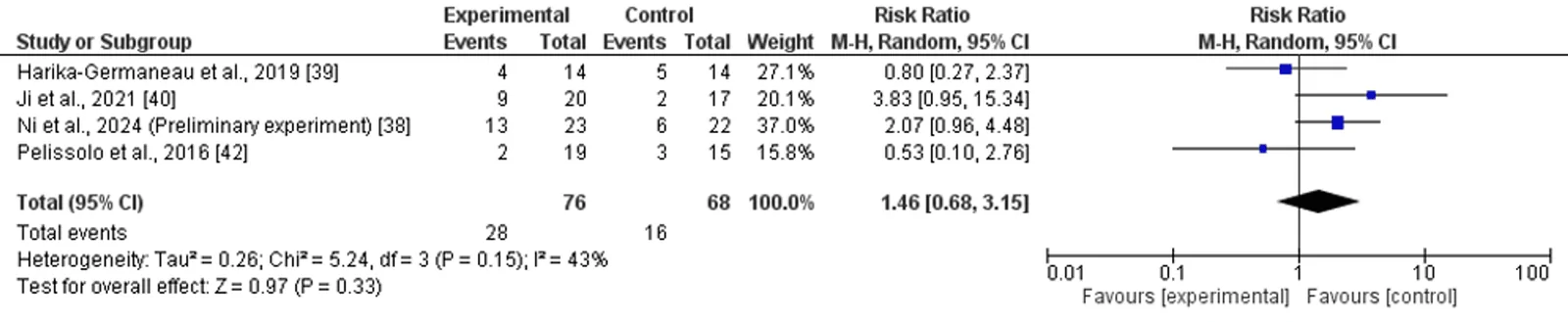
Forest plot of pooled responder rate

Narrative synthesis of non-poolable findings: Single-arm studies reported response and remission rates numerically higher than either RCT arm, though without a comparator, these cannot be attributed specifically to active stimulation. The Ni et al. outpatient extension found a 50% responder rate (16/32), again concentrated in moderate-severity patients [38]. Modirrousta et al. reported a 100% response rate among all 10 completers (range 23%-76.5% reduction) [44]. Stubbeman et al. reported OCD remission (Y-BOCS-SR ≤ 14) in 64% (9/14) of patients at a mean of 6.1 weeks, alongside comorbid depression remission in 58% (7/12) and anxiety remission in 62% (8/13) [45]. Mantovani et al. reported a 50% (4/8) responder rate at immediate post-treatment, with two initial non-responders showing delayed clinical improvement by three-month follow-up [43].

Sensitivity Analysis by Responder Definition

The four trials pooled above did not use a uniform Y-BOCS reduction threshold to define treatment response. Harika-Germaneau et al. and Pelissolo et al. [39,42] defined response as a ≥25% reduction from baseline, whereas Ji et al. [38] and Ni et al. (preliminary experiment) [40] applied a stricter ≥35% criterion (Table 2). This threshold difference was fully confounded with targeting methodology: both ≥35%-threshold trials used connectivity-personalized pre-SMA targeting based on individual subthalamic nucleus connectivity, while both ≥25%-threshold trials used fixed, non-personalized coil coordinates. A sensitivity analysis stratifying the four trials by responder definition was therefore performed.

Restricting pooling to the two ≥25%-threshold trials (Harika-Germaneau et al. [39], Pelissolo et al. [42]; 33 experimental, 29 control participants) yielded an RR of 0.71 (95% CI = 0.28, 1.75; Z = 0.75; p = .45; I² = 0%), numerically favoring sham (Figure 6).

**Figure 6:**
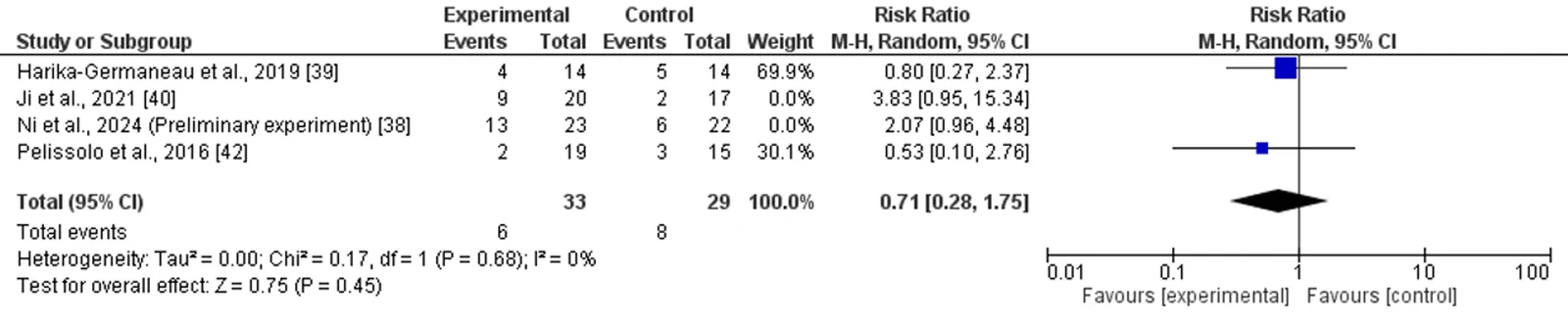
Sensitivity analysis of responder rate Restricted to trials using a ≥25% Y-BOCS reduction threshold [39,42]. Y-BOCS: Yale-Brown Obsessive Compulsive Scale.

Restricting pooling to the two ≥35%-threshold trials (Ji et al. [40], Ni et al. [38]; 43 experimental and 39 control participants) yielded an RR of 2.39 (95% CI = 1.22, 4.70; Z = 2.54; p = .01; I² = 0%), favoring active stimulation and reaching nominal significance (Figure 7). The two threshold-matched pairs thus diverged in opposite directions, with the benefit of active stimulation confined to the stricter, connectivity-personalized ≥35% subgroup. Because responder definition and targeting method were perfectly collinear across these four trials (k = 2 per subgroup), the relative contribution of threshold stringency versus targeting personalization to this divergence could not be statistically disentangled, and this comparison should be regarded as hypothesis-generating rather than confirmatory.

**Figure 7:**
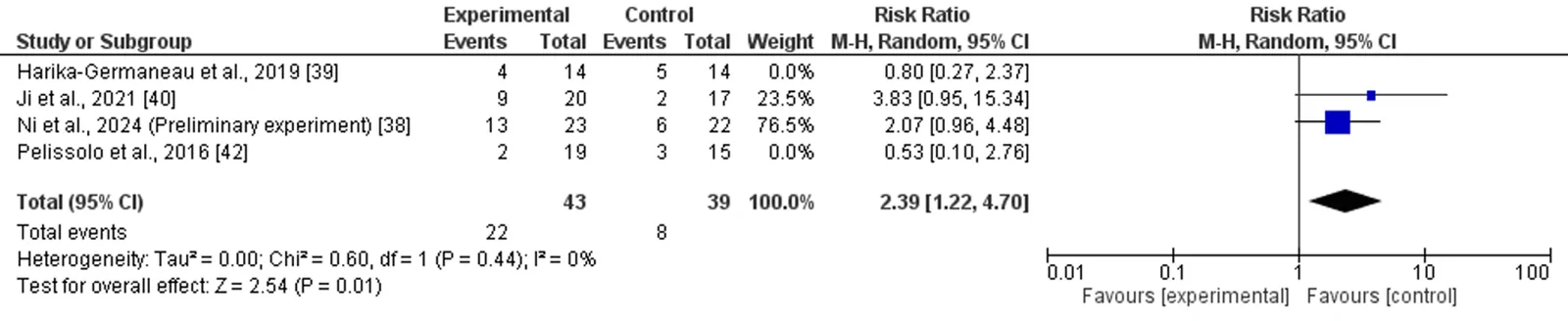
Sensitivity analysis of responder rate Restricted to trials using a ≥35% Y-BOCS reduction threshold [38,40]. Y-BOCS: Yale-Brown Obsessive Compulsive Scale.

The same threshold-matched pairing was applied to the continuous Y-BOCS mean-difference outcome. Among the two ≥25%-threshold trials, the pooled MD was 2.22 (95% CI = −0.98, 5.42; Z = 1.36; p = .17; I² = 0%), numerically favoring sham (Figure 8).

**Figure 8:**
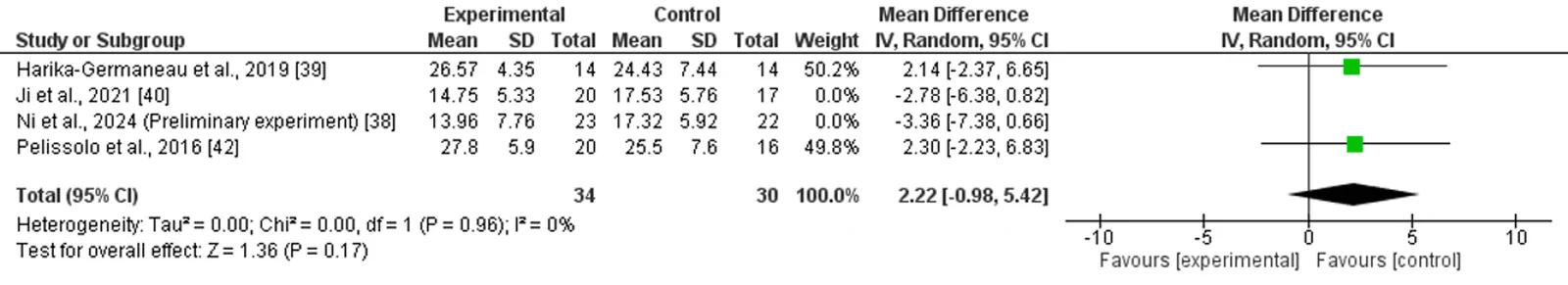
Sensitivity analysis of Y-BOCS mean difference Restricted to trials using a ≥25% responder threshold [39,42]. Y-BOCS: Yale-Brown Obsessive Compulsive Scale.

Among the two ≥35%-threshold trials, the pooled MD was −3.04 (95% CI = −5.72, −0.36; Z = 2.22; p = .03; I² = 0%), favoring active stimulation and reaching nominal significance (Figure 9). This pattern paralleled the responder-rate sensitivity analysis, with both outcomes diverging along the same targeting-methodology/threshold axis.

**Figure 9:**
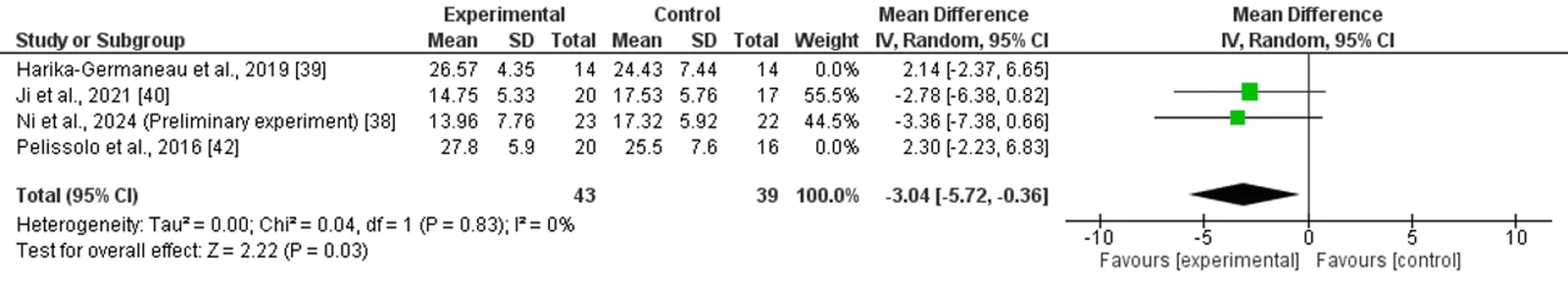
Sensitivity analysis of Y-BOCS mean difference Restricted to trials using a ≥35% responder threshold [38,40]. Y-BOCS: Yale-Brown Obsessive Compulsive Scale.

Secondary Clinical Outcomes

Secondary outcomes, including CGI, mood, anxiety, and Y-BOCS subscale scores, were reported inconsistently across trials, typically as omnibus F- or p-values rather than extractable group means, and were synthesized narratively. Across the sham-controlled trials, secondary measures were uniformly non-significant: Pelissolo et al. found no between-group differences on CGI-severity (p = .72), CGI-improvement (p = .54), the Maudsley Obsessive Compulsive Inventory, Obsessive Thoughts List, Global Assessment of Functioning, Montgomery-Åsberg Depression Rating Scale (MADRS), or Beck Anxiety Inventory (BAI) (all p = .14-.84) [42]. Harika-Germaneau et al. similarly reported no significant group effects on MADRS, the Brown Assessment of Beliefs Scale, Beck Anxiety Scale, CGI-severity, or the Hospital Anxiety and Depression Scale (all p ≥ .264) [39]. Meek et al. reported non-significant BDI (Beck Depression Inventory) and BAI change in both arms (active BDI p = .081, BAI p = .097) [41].

Y-BOCS subscale data occasionally diverged from total-score findings. In the Ni et al. preliminary experiment, the obsession subscale showed a significant Time × Group interaction (F = 4.43, p = .04) despite a non-significant total-score effect (F = 3.25, p = .08), an effect again confined to moderate-severity patients (F = 10.16, p = .004); no benefit emerged among severely ill patients in either the inpatient trial or its outpatient replication [38]. Meek et al. reported significant within-group improvement on both obsessive (F = 3.702, p = .007) and compulsive (F = 4.200, p = .003) subscales in the active but not sham arm [41]. Mantovani et al. reported significant open-label reductions on both subscales (Obsessions F = 14.147, p = .007; Compulsions F = 12.290, p = .010), and CGI improvement sustained at three-month follow-up (p = .001 vs baseline) [43]. Among uncontrolled studies, Stubbeman et al. additionally reported large within-group reductions in comorbid depressive (BDI-II: Z = 2.37, p = .0016, d = −1.7) and anxiety symptoms (BAI: Z = 3.17, p = .002, d = −1.9), though in the absence of a comparator, these findings warrant cautious interpretation [45].

Neurocognitive Outcomes

Meek et al. was the only trial to incorporate an objective, placebo-resistant cognitive outcome, using a Flanker error-monitoring task [41]. Reaction times decreased significantly over time in the active group across congruent (F = 15.595, p < .001), incongruent (F = 10.519, p = .001), and post-correct trial types (F = 15.103, p < .001), with post-hoc comparisons confirming pre-to-mid and pre-to-post reductions. The sham group showed only a modest congruent-trial reduction with no significant post-hoc effects. Error rates and post-error slowing did not differ significantly by group, suggesting a selective effect of active dACC/mPFC stimulation on processing speed rather than error-monitoring accuracy and providing an objective, less self-report-dependent signal that something changed with active stimulation; a reaction-time change alone, however, does not establish that this effect is specifically neuromodulatory in origin or that it is entirely free of non-specific or expectancy-related influences, since blinding integrity was not formally assessed in this trial.

Neuroimaging and Connectivity-Based Mechanistic Findings

Several studies reported imaging findings linking connectivity change to clinical outcome, offering mechanistic support for the personalization signal observed in the pooled analyses, though these findings were not amenable to quantitative pooling. Ji et al. derived a symptom-weighted "ideal" pre-SMA target network overlapping substantially with the ventral attention network. Similarity between an individual's connectivity profile and this ideal network significantly predicted symptom improvement in the active group (r = .60, p = .009) but not in the sham (r = −.004, p = .99), and this connectivity-outcome relationship was cross-validated in three independent external cohorts [40]. This internal cross-validation shows that the connectivity-outcome relationship generalizes across cohorts used to derive and test the model; it does not, by itself, demonstrate prospective, out-of-sample prediction of an individual, previously untreated patient's response before treatment begins. Mantovani et al. found decreased SMA connectivity with the basal ganglia, thalamus, and cerebellum following stimulation, with higher baseline Y-BOCS severity predicting both greater post-treatment connectivity and clinical non-response [43]. Ni et al. derived their pre-SMA target from individualized resting-state connectivity to the subthalamic nucleus in both the inpatient and outpatient cohorts, providing a consistent mechanistic rationale across their two positive study arms [38].

Adverse Events and Tolerability

Across all included studies, TMS was generally well tolerated, with no serious adverse events, seizures, or lasting neurological sequelae reported. The most common adverse event was mild, transient headache, occurring in a minority to roughly half of participants across trials regardless of allocation [38,39,42]. Deep dACC/mPFC stimulation via double-cone coil was associated with a broader range of transient effects, including scalp discomfort, fatigue, and, in isolated cases, transient confusion or exacerbation of pre-existing symptoms [41]; one participant in this trial withdrew due to an unpleasant pulse sensation. One participant in the Mantovani et al. pilot withdrew due to worsening depressive symptoms, judged unrelated to a specific stimulation-attributable adverse effect [43]. No trial reported treatment-emergent suicidality.

Certainty of the Evidence

Certainty of evidence was rated post hoc using GRADE (Grading of Recommendations Assessment, Development and Evaluation), applied only to comparative RCT outcomes; the four single-arm, non-randomized cohorts were instead evaluated using ROBINS-I, the appropriate tool for uncontrolled evidence. The four pooled trials were downgraded for serious risk of bias, serious inconsistency (moderate heterogeneity and directionally opposite estimates), and serious imprecision (small sample and wide null-crossing CIs); publication bias was undetected but untestable at k = 4. Meek et al. (2021), a non-pooled fifth RCT, was downgraded further for very serious imprecision because it reported only percentage change [41]. Overall certainty was rated very low across all three comparative outcomes (Table 3).

**Table 3:** GRADE summary of findings: neuroimaging-guided TMS vs. Sham for OCD ^1 ^Serious risk of bias: Based on Cochrane RoB2 ratings, most pooled trials (Pelissolo et al., Ji et al., and the Ni et al. preliminary study) were rated as having "some concerns" rather than low risk. ^2^ Serious inconsistency: Moderate heterogeneity (I² = 51% for Y-BOCS and I² = 43% for responder rate) with directionally opposite individual trial estimates (some favoring active stimulation and others favoring sham). ^3^ Serious imprecision: Small pooled sample sizes with wide confidence intervals crossing the null. ^4^ Publication bias: Undetected but untestable, as only k = 4 trials were pooled, which was too few for funnel-plot/statistical assessment. ^5^ Not serious risk of bias: Meek et al. (2021) [41] was rated as having an overall low risk of bias on RoB2. ^6^ Very serious imprecision: A single, small trial (n = 20) reported only percentage change/figure data rather than numeric means and SDs, precluding a pooled effect estimate. GRADE: Grading of Recommendations Assessment, Development and Evaluation; TMS: Transcranial magnetic stimulation; OCD: Obsessive-compulsive disorder; Y-BOCS: Yale-Brown Obsessive Compulsive Scale; RoB: Risk of bias.

Outcome	Risk of Bias	Inconsistency	Indirectness	Imprecision	Publication Bias	Overall Certainty	Effect Estimate (95% CI)
Y-BOCS total score (4 RCTs, n = 146)	Serious¹	Serious²	Not serious	Serious³	Undetected⁴	⊕⊖⊖⊖ Very low	MD = −0.64 (−3.60 to 2.32)
Responder rate (4 RCTs, n = 144)	Serious¹	Serious²	Not serious	Serious³	Undetected⁴	⊕⊖⊖⊖ Very low	RR = 1.46 (0.68 to 3.15)
Y-BOCS total score (1 RCT, n = 20; Meek et al., 2021 [41])	Not serious⁵	N/A (single study)	Not serious	Very serious⁶	Undetected	⊕⊖⊖⊖ Very low	Not poolable – % change only (28.0% active vs. 11.7% sham at 1 month)

Discussion

This systematic review and meta-analysis sought to determine whether the degree of neuroimaging-based targeting personalization, rather than stimulation frequency or anatomical target alone, moderates clinical response to TMS in treatment-resistant OCD. Across five sham-controlled RCTs and four open-label studies, the pooled quantitative analyses of Y-BOCS total score (MD = −0.64, 95% CI = −3.60, 2.32) and responder rate (RR = 1.46, 95% CI = 0.68, 3.15) both failed to reach statistical significance. However, a consistent and clinically meaningful pattern emerged beneath this null pooled estimate: trials employing individually connectivity-personalized pre-SMA targeting [38,40] trended toward benefit on both outcomes, while trials using fixed-coordinate or structurally neuronavigated targeting without individualized connectivity data [39,42] trended numerically toward sham. This directional split, most pronounced for responder rate, where the connectivity-personalized trials showed RRs of 3.83 [40] and 2.07 [38] against fixed-coordinate RRs of 0.80 and 0.53, is consistent with the hypothesis that targeting precision, not merely region or frequency, is an important moderator of clinical response, though the small number of pooled trials leaves this as a signal rather than a confirmed effect. Because targeting methodology was not itself randomized and no formal subgroup meta-regression or interaction test was performed, and because the connectivity-personalized and fixed-coordinate trials also differed in stimulation modality (cTBS versus rTMS), coil type, and patient populations, this directional split is confounded by factors beyond targeting method alone. It should therefore be interpreted strictly as a hypothesis-generating, exploratory pattern rather than as evidence that targeting personalization is itself the causal driver of the observed difference.

This finding stands in productive contrast to the three most relevant prior meta-analyses. Fitzsimmons et al., pooling 21 sham-controlled RCTs without regard to targeting methodology, identified three efficacious protocols, low-frequency pre-SMA stimulation, high-frequency bilateral DLPFC stimulation, and low-frequency right DLPFC stimulation, with an overall pairwise effect of Hedges' g = −0.502, considerably larger than the null estimate observed in our imaging-guided subset [31]. This divergence is instructive: because Fitzsimmons et al.'s network clustered trials purely by frequency-location combination, it could not distinguish whether the pre-SMA protocols they identified as efficacious were driven by well-personalized trials, fixed-coordinate trials, or an average of both, precisely the ambiguity our review was designed to resolve. Liu et al., who deliberately restricted their pool to non-neuronavigated, low-frequency surface-coil protocols, reported a near-identical null pooled estimate (MD = −0.69, 95% CI (−1.75, 0.38)) to our own (MD = −0.64), despite using a substantially larger sample (14 RCTs, N = 460) than our four-trial pool [32]. The convergence of these two independent null estimates is notable: it suggests that when trials using minimally individualized targeting are considered in isolation, whether by explicit design [32] or by chance composition, as two of our four pooled trials used standard-coordinate targeting, the evidence for benefit disappears. Liu et al. (2025) explicitly flagged this as an open question, writing that "whether neuronavigation and cortical field verification can bridge this performance gap requires adequately powered trials designed for direct protocol comparison" [32].

Our review, by explicitly stratifying trials along the personalization gradient rather than treating neuronavigation as a binary or incidental feature, provides an initial answer: the two connectivity-personalized trials in our pool numerically outperformed the two fixed-coordinate trials on both primary outcomes, offering preliminary, though underpowered, support that personalization, not navigation per se, is the operative variable, a hypothesis that requires confirmation in adequately powered, prospectively randomized head-to-head trials rather than the retrospective stratification used here. Khosravi et al., the largest and most recent of the three prior reviews (47 RCTs), reached a similarly cautious conclusion, noting that studies "incorporating neurobiological predictors such as neuroimaging or genetic polymorphisms suggest potential for enhanced outcomes through personalized treatment strategies" but did not formally test this as a moderator. Our review offers an exploratory, descriptive comparison consistent with their narrative observation; given the small number of pooled trials and the absence of a formal subgroup meta-regression or interaction test, it does not constitute a direct quantitative test of personalization as a moderator [14].

Beyond the primary outcome, the Y-BOCS subscale and secondary-outcome data offer converging support for a severity-dependent, connectivity-mediated response pattern rather than a uniform treatment effect. In the Ni et al. preliminary experiment, the obsession subscale showed a significant Time × Group interaction (F = 4.43, p = .04) despite a non-significant total-score effect, with benefit confined to moderate-severity patients (F = 10.16, p = .004) and absent in severely ill patients across both the inpatient trial and its outpatient extension [38]. This severity-response gradient was independently foreshadowed in the small Mantovani et al. pilot [43], where the two non-responders also had the highest baseline Y-BOCS scores, and higher baseline severity predicted greater residual post-treatment hyperconnectivity between SMA and subcortical regions. This finding directly parallels the connectivity-outcome relationship formally validated by Ji et al. [40], whose symptom-weighted "ideal" pre-SMA target network (overlapping substantially with the ventral attention network) predicted individual symptom improvement in the active group (r = .60, p = .009) but not the sham group (r = −.004, p = .99). This model was cross-validated in three independent external cohorts [40,43]. Taken together, these three independently derived findings, a formal RCT interaction effect [38], a connectivity-outcome correlation validated across cohorts [40], and a converging observational signal from an open-label pilot [43], constitute a triangulated mechanistic narrative: individualized connectivity data appears to identify which patients, and which specific target coordinates within a nominal region, are most likely to benefit, and baseline illness severity may itself constrain the ceiling of that benefit. It is important to note that none of these three findings demonstrates prospective, out-of-sample prediction of an individual patient's treatment response from baseline connectivity data in a previously unseen cohort. The cross-validation reported by Ji et al. [40] was performed within cohorts already used to develop and test the target model. These findings are therefore better characterized as establishing mechanistic plausibility for a connectivity-response relationship than as demonstrating that connectivity-guided targeting can currently be used prospectively, in routine clinical practice, to identify which individual patients will respond.

Secondary clinical outcomes (CGI, mood, and anxiety measures) were uniformly non-significant across the sham-controlled trials [39,41,42], a pattern broadly consistent with Fitzsimmons et al.'s observation that rTMS effects on comorbid depression, while present, are typically modest relative to the primary OCD outcome [31]. The one trial incorporating an objective, placebo-resistant neurocognitive outcome, Meek et al.'s Flanker task, found selective improvement in reaction time but not error-monitoring accuracy in the active group, offering a rare non-self-reported corroboration that some treatment-related change occurred. However, as noted above, this reaction-time finding alone does not establish that the effect is specifically neuromodulatory or entirely free of non-specific/expectancy influences, a form of evidence largely absent from the outcome sets of the three prior reviews [41]. Across all nine study arms, TMS was well tolerated, with no serious adverse events, seizures, or treatment-emergent suicidality reported, and adverse-event profiles differed primarily by coil type (double-cone deep stimulation producing more scalp discomfort and transient cognitive effects than figure-eight surface coils) rather than by targeting methodology, indicating that the safety profile of neuroimaging-guided TMS is not meaningfully altered by the addition of individualized targeting, even as its efficacy signal may be.

This review has several strengths. It is a rare systematic review of TMS for OCD to formally stratify trials along a continuous personalization gradient rather than treating neuronavigation as a binary or incidental trial feature, directly addressing the gap. Study selection and data extraction were conducted independently and in duplicate; risk of bias was assessed using current, domain-appropriate Cochrane tools (RoB2 for RCTs and ROBINS-I for non-randomized studies) rather than a single generic scale; and non-poolable and non-randomized evidence was synthesized narratively rather than discarded, preserving clinically relevant signal that a purely quantitative pooling approach would have omitted. The inclusion of an objective neurocognitive outcome (the Meek et al.'s Flanker task) and cross-validated connectivity-outcome data allowed the review to move beyond self-reported Y-BOCS scores alone when evaluating mechanistic plausibility.

For clinicians, these findings do not currently support preferring connectivity-personalized targeting over standard-coordinate neuronavigation on grounds of proven superior efficacy; both remain reasonable options within a shared decision-making framework, and coil/targeting choice may instead reasonably be guided by cost, access to imaging and connectivity-processing infrastructure, and patient tolerability. For researchers, the consistent directional split observed across independent outcomes (Y-BOCS total score, responder rate, and the obsession subscale) despite non-significant pooled estimates argues for adequately powered, head-to-head trials that randomize patients between fixed-coordinate and connectivity-personalized targeting within the same study, holding coil type, frequency, and population constant, as the only design capable of formally testing personalization as a moderator rather than inferring it from between-trial comparisons.

Priority directions for future work include the following: (1) adequately powered, multi-site RCTs directly randomizing treatment-resistant OCD patients to fixed-coordinate neuronavigated versus individually connectivity-personalized pre-SMA targeting, powered for a formal interaction/subgroup test rather than a between-trial comparison; (2) harmonization of responder-rate thresholds (e.g., prospective, pre-registered adoption of a single Y-BOCS reduction criterion) across future trials to reduce definitional heterogeneity of the kind identified in this review; (3) prospective, out-of-sample validation of connectivity-derived ''ideal target" models of the kind proposed by Fitzsimmons et al. [31] in genuinely independent, previously unseen cohorts to test whether they can identify individual responders before treatment rather than only correlate with outcome after the fact; (4) inclusion of the currently unavailable Mukherjee et al. accelerated cTBS trial [46] once its full text becomes accessible, with re-pooling of the primary outcomes; and (5) a formal GRADE-based certainty-of-evidence rating and prospective PROSPERO registration for any future update of this review.

Limitations

This review has several limitations. First, the pooled meta-analytic sample was small (four RCTs for Y-BOCS and four for responder rate), limiting statistical power and precluding formal subgroup meta-regression to test targeting personalization as a moderator; the observed connectivity-personalized versus fixed-coordinate split should therefore be interpreted as hypothesis-generating rather than confirmatory. Second, a potentially eligible trial, Mukherjee et al.'s randomized sham-controlled study of adjunctive neuronavigated accelerated cTBS in OCD, could not be included because its full text was unavailable, introducing possible selection bias into the evidence base [46]. Third, this review was restricted to English-language publications, which may have excluded relevant non-English studies of neuroimaging-guided TMS in OCD and introduces the possibility of language/geographic bias in the evidence base synthesized here. Fourth, this review was not registered in PROSPERO, precluding a priori protocol transparency. Fifth, four of nine included study arms were open-label or retrospective with no comparator, all judged at serious risk of bias via ROBINS-I, restricting their contribution to narrative rather than quantitative synthesis. Finally, secondary and neuroimaging outcomes were reported too inconsistently (varying scales, omnibus statistics, and figure-only data) to permit quantitative pooling, limiting the strength of conclusions drawn about mechanism and secondary clinical benefit.

## Conclusions

Across the pooled randomized trials, neuroimaging-guided TMS did not demonstrate a statistically significant overall benefit over sham for either Y-BOCS total score or responder rate in treatment-resistant OCD, and considerable heterogeneity was observed across studies. However, a consistent pattern emerged when trials were examined by targeting methodology: those using individually connectivity-personalized pre-SMA targeting trended toward clinical benefit, whereas trials relying on fixed-coordinate or standard-anatomical neuronavigation trended toward null or sham-favoring results. This pattern was reinforced by narrative synthesis of non-randomized and non-poolable studies, which reported larger within-group symptom reductions, particularly among patients with moderate severity, and by mechanistic neuroimaging findings linking individualized connectivity profiles to symptom improvement. TMS was well tolerated across all targeting approaches, with no serious adverse events reported. Taken together, these findings suggest that the degree of imaging-based individualization, rather than stimulation frequency or anatomical target alone, may be an important driver of clinical outcome in OCD. However, this remains a hypothesis-generating observation drawn from an underpowered, non-randomized comparison of targeting strategies and should not be interpreted as a confirmed causal finding. However, the current evidence base remains limited by small sample sizes, inconsistent outcome reporting, and an underpowered pooled sample, precluding formal subgroup meta-regression. Adequately powered trials directly comparing standard-coordinate neuronavigation with individually connectivity-personalized targeting are needed to confirm whether targeting personalization can meaningfully improve treatment outcomes for patients with treatment-resistant OCD.

## Appendices

Full search strategy by database

**Table 4:** Search strategy by database cTBS: Continuous theta-burst stimulation; DTI: Diffusion tensor imaging; dTMS: Deep transcranial magnetic stimulation; EEG: Electroencephalography; fMRI: Functional magnetic resonance imaging; iTBS: Intermittent theta-burst stimulation; MEG: Magnetoencephalography; MRI: Magnetic resonance imaging; OCD: Obsessive-compulsive disorder; PET: Positron emission tomography; rTMS: Repetitive transcranial magnetic stimulation; rs-fMRI: Resting-state functional magnetic resonance imaging; SPECT: Single-photon emission computed tomography; TMS: Transcranial magnetic stimulation.

Database	Search Date	Full Search String	Filters Applied	Records Identified
PubMed	Database inception–July 14, 2026	("Obsessive-Compulsive Disorder"[Mesh] OR OCD OR "obsessive compulsive disorder") AND ("Transcranial Magnetic Stimulation"[Mesh] OR "transcranial magnetic stimulation" OR TMS OR rTMS OR dTMS OR cTBS OR iTBS OR theta burst OR "deep transcranial magnetic stimulation") AND (neuroimaging OR imaging OR MRI OR magnetic resonance imaging OR fMRI OR "functional MRI" OR rs-fMRI OR "resting-state fMRI" OR PET OR "positron emission tomography" OR SPECT OR EEG OR MEG OR neuronavigation OR neuronavigated OR "image-guided" OR "MRI-guided" OR connectivity-guided OR connectome OR tractography OR DTI OR "functional connectivity")	None	185
Cochrane Library	Database inception–July 14, 2026	("obsessive compulsive disorder" OR OCD) AND ("transcranial magnetic stimulation" OR TMS OR rTMS OR cTBS OR iTBS OR dTMS OR "deep transcranial magnetic stimulation" OR "deep TMS") AND (neuroimaging OR imaging OR MRI OR fMRI OR rs-fMRI OR "resting-state fMRI" OR PET OR SPECT OR EEG OR MEG OR neuronavigation OR neuronavigated OR navigated OR "MRI-guided" OR "image-guided" OR connectome OR connectivity-guided OR "functional connectivity" OR tractography OR DTI)	None	126
ScienceDirect	Database inception–July 14, 2026	("obsessive-compulsive disorder" OR OCD) AND ("transcranial magnetic stimulation" OR rTMS) AND (neuronavigat OR "MRI-guided") AND ("functional connectivity" OR connectome)	None	79
Total				390
